# The Viability and Protein Expression of Beijing/W Lineage *Mycobacterium tuberculosis* Circulating in Xinjiang, China

**DOI:** 10.1007/s00284-015-0776-z

**Published:** 2015-02-06

**Authors:** Feng Li, Hua Li, Wei-ze Zuo, Ligu Mi, Xian Wang, Yuanzhi Wang, Hong Wang, Aiping Shen, Shuaili Cao, Li Yuan

**Affiliations:** 1 Department of Microbiology and Immunology, Xinjiang Laboratory of Endemic and Ethnic Disease, Shihezi University, Shihezi, 832000 China; 2 The First Hospital of Shihezi University, Shihezi, 832000 China

## Abstract

Beijing/W lineage strains of *Mycobacterium tuberculosis* spread faster than other strains, tend to be more virulent and frequently associated with drug resistance. In this study, to distinguish the characteristics of Beijing/W lineage and non-Beijing/W lineage *M. tuberculosis*, we assessed the growth between the two groups under conditions of hypoxia, nutrient starvation, and intracellular growth in murine macrophages. We also examined the DNA, RNA, and protein levels of 5 major *M. tuberculosis* proteins, including HspX, Hsp65, 38 kDa, Ag85B, and MPT64 of the different types of strains by sequencing, quantitative RT-PCR, and Western blotting. The results showed that Beijing/W and non-Beijing/W lineage strains of *M. tuberculosis* have similar viability in ex vivo culture but differ in their ability to survive within macrophages, and the intracellular viability of the Beijing/W lineage strains was significantly more than the viability of the non-Beijing/W lineage strains at 2, 3, and 5 days after infection (*P* < 0.05). *Psts1* and *fbpB* were expressed at statistically lower levels in Beijing/W lineage strains in their mRNA expression levels (*P* < 0.05). The expression of their corresponding 38 kDa and Ag85B was lower in the Beijing/W lineage strains than the non-Beijing/W lineage strains (*P* < 0.05). The expression of HspX and Hsp65 was higher in the Beijing/W lineage strains in their protein expression levels at 24 h after infection of RAW264.7 macrophages (*P* < 0.05). In conclusion, the increased viability of the Beijing/W lineage strains might be related to the expression levels of these proteins.

## Introduction

Tuberculosis (TB) is a chronic infectious disease caused by *Mycobacterium tuberculosis*, which poses a serious threat to human health. The prevalence of *M. tuberculosis* is subject to regional differences. According to WHO [17] survey report, the largest number of new TB cases occurs in Asia, accounting for 60 % of the new cases globally. China is undergoing the world’s second largest TB epidemic, and within China, Xinjiang is the highest incidence area.

The Beijing/W lineage strains comprise a unique genetic lineage of *M. tuberculosis*, accounting for 13 % of global isolates [1]. These strains spread faster than other strains, tend to be more virulent [20] and frequently associated with drug resistance [5, 10], but what factors make Beijing/W lineage got the above characteristics are unclear. The characteristics of the Beijing/W lineage strains are thought to be related to changes in the intrinsic expression of specific molecules. So, we choose the 5 proteins (HspX, Hsp65, 38 kDa, Ag85B, and MPT64), in which the 5 proteins were confirmed with *M. tuberculosis* virulence or host immune response against *M. tuberculosis* by studies. We want to know that the 5 protein whether there were difference between Beijing/W lineage and non-Beijing/W lineage *M. tuberculosis* strains at gene level, mRNA level, and protein levels. HspX coded by gene *hspX* was an important membrane antigen of *M. tuberculosis*. HspX was overexpressed and became the main protein in the latent infection. It played an important role in the viability and stability of *M. tuberculosis* and was related to the function of molecular chaperone. The secreted protein Hsp65 coded by gene *gorEL2* was a member of the Hsp60 family. When *M. tuberculosis* got into the host, Hsp65 would be the important protective antigen of the body against the invasion. It was highly conserved and had strong immunogenicity. 38 kDa coded by gene psts1 is a protein related to the phosphorous metabolism and could mediate the apoptosis of macrophages. Ag85B coded by gene fbpB has the protective immune function and can induce strong humoral and cellular immune response. It has the acid transferase activity to catalyze the synthesis of cord factor so it plays an important role in the final stage of cell wall synthesis and maintaining its integrity. Mpt64 coded by gene mpt64 is an important secreted protein in the early culture filtrate of MTB and has strong immunogenicity. One study showed that the overexpression of MPT64 could inhibit the apoptosis of macrophage in host, so the motility of bacterium was induced. To identify factors that may explain differences among strains, we examined the DNA, mRNA, and protein levels of 5 major *M. tuberculosis* proteins, including HspX, Hsp65, 38 kDa, Ag85B, and MPT64. Our results help to characterize factors that may contribute to the enhanced virulence of the Beijing/W lineage strains of *M. tuberculosis* in Xinjiang.

## Methods

### Mycobacterial Specimens

This study included 30 Beijing/W lineage and 30 non-Beijing/W lineage *M. tuberculosis* isolates collected from the sputum of patients with TB. Informed consent was obtained from participants prior to initiation of the study, and approval was granted from the medical ethics committee at the first hospital of Shihezi University.

### Strain Isolation and Identification

Sputum samples were isolated and cultured on Lowenstein–Jensen (L–J) culture media. A preliminary identification of mycobacterial species was conducted by applying differential Para Nitro Benzoic Acid (PNB) and Thiophene-2-Carboxylic Acid Hydrazide (TCH) media [4]. Subsequently, *M. tuberculosis* identification was performed based on the 16 s rRNA and MTP40 sequences [9]. Beijing/W lineage strains were identified by PCR assay for deletions in region of difference 105(RD105) [15]. Strain isolation and identification were completed at the Ministry of Education Key Laboratory of Xinjiang Endemic and Ethnic Disease.

### Measurement of Strain Viability Under Various Conditions

*Mycobacterium tuberculosis* H37Rv and clinical isolates taken from L–J medium slants were inoculated into Middle-brook 7H9 medium containing 0.05 % Tween 80 supplemented with 10 % ADC (Difco) and cultured for 10–14 days at 37 °C with shaking at 12 h intervals. Bacterial suspensions adjusted to the same turbidity were transferred at 10^6^ CFU/mL to tubes under the following 3 growth conditions: (1) Standard culture: The bacilli were cultured in 7H9 medium. Breathable silicone plugs separated a 1:2 air-to-medium volume ratio, and methylene blue was added to a final concentration of 1.5 μg/mL as an indicator of the presence or absence of an oxygen. (2) Hypoxic culture: The bacilli were cultured in 7H9 medium with methylene blue; however, tube plugs were replaced with airtight rubber stoppers and sealed with sealing film. (3) Nutrient starvation culture: The bacteria suspension was centrifuged at 10,000 rpm for 5 min, washed twice with PBS, and then resuspended in PBS at 37 °C. At different time points (2, 4, 7, 10 days) of growth under each of the three culture conditions, equal amount of bacteria was inoculated into L–J medium after being diluted tenfold serially and cultured at 37 °C for 4 weeks to observe the growth and colony count.

### Measurement of Intracellular Mycobacterial Growth

RAW264.7 macrophages were cultured at 1 × 10^6^ cells/mL in RPMI 1,640 with 10 % fetal bovine serum at 37 °C in a 5 % CO_2_ incubator. 10^7^ *M*. *tuberculosis* in the logarithmic growth phase was added to 10^6^ macrophages, and the mixture was cultured in RPMI 1,640 with 10 % serum in a 6-well culture plate. After 4 h, the cells were washed 3 times with warm RPMI in order to remove the *M. tuberculosis* that had not been engulfed by the macrophages, and 2 mL RPMI 1,640 with 10 % serum was added. This point was considered time zero of bacterial infection. At different time points after time zero (0.5, 1, 1.5, 2, 3, 5 days), the culture medium was discarded, and 250 μL RIPA lysis buffer with 1 % PMSF was added for 30 min. After being diluted tenfold, equal amount of lysates was inoculated into L–J medium and cultured at 37 °C for 4 weeks to observe the growth and colony count.

### Genomic DNA Extraction and Sequencing

Scraped colonies growing on L–J medium were suspended in 500 μL of distilled water and inactivated at 85 °C for 30 min. The bacteria were then centrifuged at 8,000 rpm for 15 min, and the pellets were resuspended in 400 μL of TE (pH 8.3), boiled for 30 min, and centrifuged at 12,000 rpm for 5 min. Supernatants were collected and stored at −20 °C until further use. Specific primers were designed using primer 5.0 software based on the sequences of hspX, groEL2, psts1, fbpB, and mpt64 in GeneBank (Table 1). PCR products were electrophoretically separated on 1 % agarose gels and sequenced.

**Table 1:** Primers for gene sequencing

Gene	Sequence (5′–3′)	Amplicon size (bp)	PCR annealing temperature (°C)
hspX	F: 5′-CGCGGATCCATGGCCACCACCCTTC-3′	453	62
	R: 5′-CCCAAGCTTTCAGTTGGTGGACCGG-3′		
groEL2	F: 5′-ATGGCCAAGACAATTGCGTA-3′	1,623	53
	R: 5′-TCAGAAATCCATGCCACCC-3′		
psts1	F: 5′-AAATGAACCTGGTCGAGGAAC-3′	848	60
	R: 5′-TTTCACGAGAGGTATCCGAAC-3′		
fbpB	F: 5′-ATGACAGACGTGAGCCGAA-3′	970	56
	R: 5′-CGCCTAACGAACTCTGCAG-3′		
mpt64	F: 5′-AAGATCTTCATGCTGGTCACG-3′	678	56
	R: 5′-CTAGGCCAGCATCGAGTCG-3′		

### Quantitative Real Time-PCR Analysis of *M. tuberculosis*

Bacteria suspension (3 mL) at logarithmic phase in 7H9 medium was collected and centrifuged at 8,000 rpm for 5 min. The supernatant was discarded, and the sediment was washed 3 times with PBS. Total bacterial RNA was extracted according to the manufacturer’s instructions (bacterial RNA extraction kit; Omega). The concentration and purity were assayed with nucleic acid protein detector, and equal amounts of RNA in each sample were used for reverse transcription. Total RNA was denaturalized at 65 °C for 5 min and then placed on ice immediately. Reverse transcription was performed using 0.5 μL primers, 0.5 μL reverse transcriptase, 2.0 μL reaction buffer, 1.0 μL RNA, and 6.0 μL RNase-free H_2_O. After reverse transcription, the cDNA was stored at −20 °C.

**Fig. 1:**
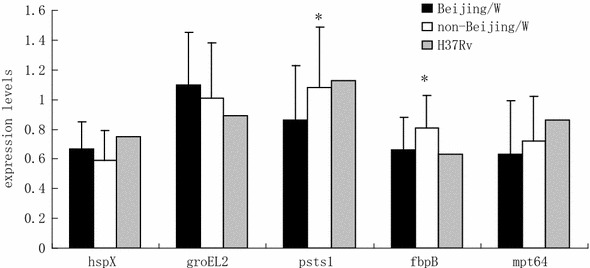
The mRNA expression level in *M. tuberculosis. Asterisk* the difference between Beijing/W lineage and non-Beijing/W lineage strains is significant (*P* < 0.05)

The specific primers for *hspX*, *groEL2*, *psts1*, *fbpB*, *mpt64,* and the reference *gene SigA* were designed by primer 5.0 software for use in quantitative RT-PCR (Table 2). The quantitative RT-PCR mixture consisted of 2 μL cDNA template, 10 μL SYBR Premix, 7 μL ddH_2_O, and 0.5 μl of each primer (10 pmol/μL). Each gene of each sample was assessed in triplicate. The relative expression of *hspX*, *groEL2*, *psts1*, *fbpB*, *mpt64*, and the reference gene *SigA* was calculated by the 2^−ΔΔCt^ value (Fig. 1).

**Table 2:** Primers for qRT-PCR

Gene	Sequence (5′–3′)	Amplicon size (bp)	PCR annealing temperature (°C)
hspX	F: 5′-CGACAAGGACGTCGACATTA-3′	173	60
	R: 5′-CCTTGTCGTAGGTGGCCTTA-3′		
groEL2	F: 5′-GTAGTGGCTTACCCGTTCCTG-3′	195	60
	R: 5′-TGGGCACCGAGTTGGAGT-3′		
psts1	F: 5′-GATGTTCATCAGCCCCTTGT-3′	187	62
	R: 5′-CTACCCGCTGTTCAACCTGT-3′		
fbpB	F: 5′-GAACAACTCACCTGCGGTTT-3′	215	62
	R: 5′-TCAGGAAGGTTTCCCACTTG-3′		
mpt64	F: 5′-TCGTTTTGCTCTGTTGTTCG-3′	185	62
	R: 5′-CGTCTGGGCGATGTAATTTT-3′		
SigA	F: 5′-TCGAGGTGATCAACAAGCTG-3′	254	62
	R: 5′-CTGCAGCAAAGTGAAGGACA-3′

### Western Blotting

After 24 h infection, RAW264.7 macrophages were collected and centrifuged at 2,000 rpm for 10 min. The supernatants were discarded, and the cells were washed 3 times with PBS. For each 10^6^ cells, 250 μL of RIPA lysis buffer with 1 % PMSF was added for 30 min, and then cells were ultrasonically broken for 1 min at 70 % amplitude, 1 s ultrasonic time, 1 s interval, and work 30 times and centrifuged at 4 °C and 14,000 rpm for 25 min. Supernatants were collected and stored at −80 °C. The concentration and purity of total proteins were assayed with a nucleic acid protein detector, and the protein concentration of each sample was equalized for use in Western blotting.

Proteins were denaturalized at 100 °C for 5 min, and 50 μg samples were separated by SDS-PAGE gel electrophoresis (5 % stacking gel and 12 % separation gel) and transferred by the semi-dry transfer method to PVDF membranes. The membranes were sealed in 5 % skim milk for 2 h, incubated overnight at 4 °C in primary antibody (HspX monoclonal antibody diluted 1:2,000, Hsp65 monoclonal antibody diluted 1:100, 38 kDa monoclonal antibody diluted 1:5,000, Ag85B polyclonal antibody diluted 1:5,000, MPT64 polyclonal antibody diluted 1:1,000, or β-actin monoclonal antibody diluted 1:1,000 as internal control). Blots were washed for 10 min in TBST 5 times, incubated at room temperature for 1 h in IgG-HRP, washed in TBST, and exposed using an ECL chemiluminescent kit. Gray values of the target and control protein bands were determined using a Quantity One Analyzer, and average ratios of proteins relative to β-actin expression were calculated.

## Results

### Beijing/W and Non-Beijing/W Lineage Strains of *M. tuberculosis* Have Similar Viability in Ex Vivo Culture

To determine whether Beijing/W and non-Beijing/W lineage strains differ in their growth properties, we cultured 30 isolates of each strain type from TB patients in Xinjiang and assessed their growth following culture under control, hypoxia, and nutrient depletion conditions. The color of the control cultures remained a pale blue color due to the methylene blue indicator, whereas the hypoxia medium became colorless after 6 days, verifying appropriate depletion of oxygen. After different time points of culture (2, 4, 7, 10 days) under the three different conditions, serial dilutions of the bacteria were plated, and resulting colony numbers were assessed after 4 weeks of growth. The effects of hypoxia in inhibiting cell viability were apparent in 7 and 10 days cultures, and the effects of nutrient depletion were clear at all culture times; however, there was no statistical difference between the Beijing/W and non-Beijing/W lineage strains in viability under any of the three culture conditions (*P* > 0.05; Table 3). These results suggest that the Beijing/W and non-Beijing/W lineage strains do not differ significantly in their ability to grow in the absence of a host cell.

**Table 3:** The numbers of living bacterium after different incubation times under various conditions

Time point (days)	The logarithm of colonies (Log10 CFU/mL)								
	Control			Hypoxia			Nutrient starvation		
	Beijing/W	Non-Beijing/W	H37Rv	Beijing/W	Non-Beijing/W	H37Rv	Beijing/W	Non-Beijing/W	H37Rv
2	6.99 ± 0.14	6.97 ± 0.04	6.87	6.89 ± 0.35	6.89 ± 0.18	6.57	5.43 ± 0.07	5.39 ± 0.07	5.36
4	7.10 ± 0.29	7.09 ± 0.33	6.96	7.03 ± 0.59	7.05 ± 0.27	6.88	5.38 ± 0.05	5.26 ± 0.16	5.15
7	7.59 ± 0.46	7.45 ± 0.56	7.69	6.98 ± 0.27	6.81 ± 0.39	6.65	5.07 ± 0.10	5.07 ± 0.10	5.06
10	7.85 ± 0.70	7.86 ± 0.34	7.93	6.84 ± 0.52	6.64 ± 0.64	6.38	5.03 ± 0.06	4.84 ± 0.08	4.67

### Beijing/W and Non-Beijing/W Lineage Strains of *M. tuberculosis* Differ in Their Ability to Survive Within Macrophages

*Mycobacterium tuberculosis* is a typical intracellular pathogen, and macrophage is a major host cell of *M. tuberculosis* in the body. Macrophage with powerful phagocytosis can protect the host against *M. tuberculosis* infection. However, in the long process of interaction with the host macrophages, *M. tuberculosis* formed gradually a variety of effective strategies to evade being killed, to survive, and to proliferate in the host macrophages. To determine whether Beijing/W and non-Beijing/W lineage strains differ in their capacity to infect or thrive within macrophages, we infected RAW264.7 macrophages with different strains of *M. tuberculosis* and assessed the amount of viable intracellular bacteria by plating colonies following a time course of incubation. The H37Rv strain of *M*. *tuberculosis* was also tested as a comparison point. The different types of strains produced comparable numbers of colonies within the first 1.5 days, indicating similar rates of infection. However, the Beijing/W lineage strains produced more viable colonies than the non-Beijing/W lineage strains and H37Rv at 2, 3, and 5 days after infection (*P* < 0.05; Table 4). These results suggest that the Beijing/W lineage strains have a similar capacity to grow in culture and a similar rate of infectivity, but that they have a greater capacity to thrive within macrophages. This greater capacity could underlie their overall increased prevalence and virulence [20].

**Table 4:** Viability of MTB in the macrophages

Time point (days)	The logarithm of colonies (Log10 CFU/well)		
	Beijing/W	Non-Beijing/W	H37Rv
0.5	2.15 ± 0.04	2.24 ± 0.06	2.16
1	2.42 ± 0.13	2.36 ± 0.23	2.33
1.5	2.52 ± 0.16	2.47 ± 0.12	2.49
2	2.64 ± 0.32	2.18 ± 0.27	2.36
3	2.42 ± 0.23	2.12 ± 0.18	2.22
5	1.95 ± 0.08	1.24 ± 0.11	1.39

### The Sequences of Key Genes from the Beijing/W and Non-Beijing/W Lineage Strains are Identical

To determine whether differences in intracellular grow by the Beijing/W and non-Beijing/W lineage strains may be related to divergent sequences of key genes, we sequenced 5 genes *hspX*, *groEL2*, *psts1*, *fbpB,* and *mpt64*, which encode the proteins HspX, Hsp65, 38 kDa, Ag85B, and MPT64, respectively. We used the DNAMAN program to compare the gene sequences from Beijing/W and non-Beijing/W lineage strains with those of the standard H37Rv strain. No nucleotide variations were identified in hspX, groEL2, psts1, or mpt64. Furthermore, a C to A base variation was found at 713 bp of fbpB (incidence 42 %); however, because of codon degeneracy, the amino acid in this position did not change, and the mutation was found in both Beijing/W lineage and non-Beijing/W lineage strains. These results suggest that variation in the sequence of the 5 genes cannot explain the differing growth properties of Beijing/W and non-Beijing/W lineage strains of *M*. *tuberculosis*.

### Beijing/W and Non-Beijing/W Lineage Strains Vary in Their mRNA and Protein Expression Levels

**Fig. 2:**
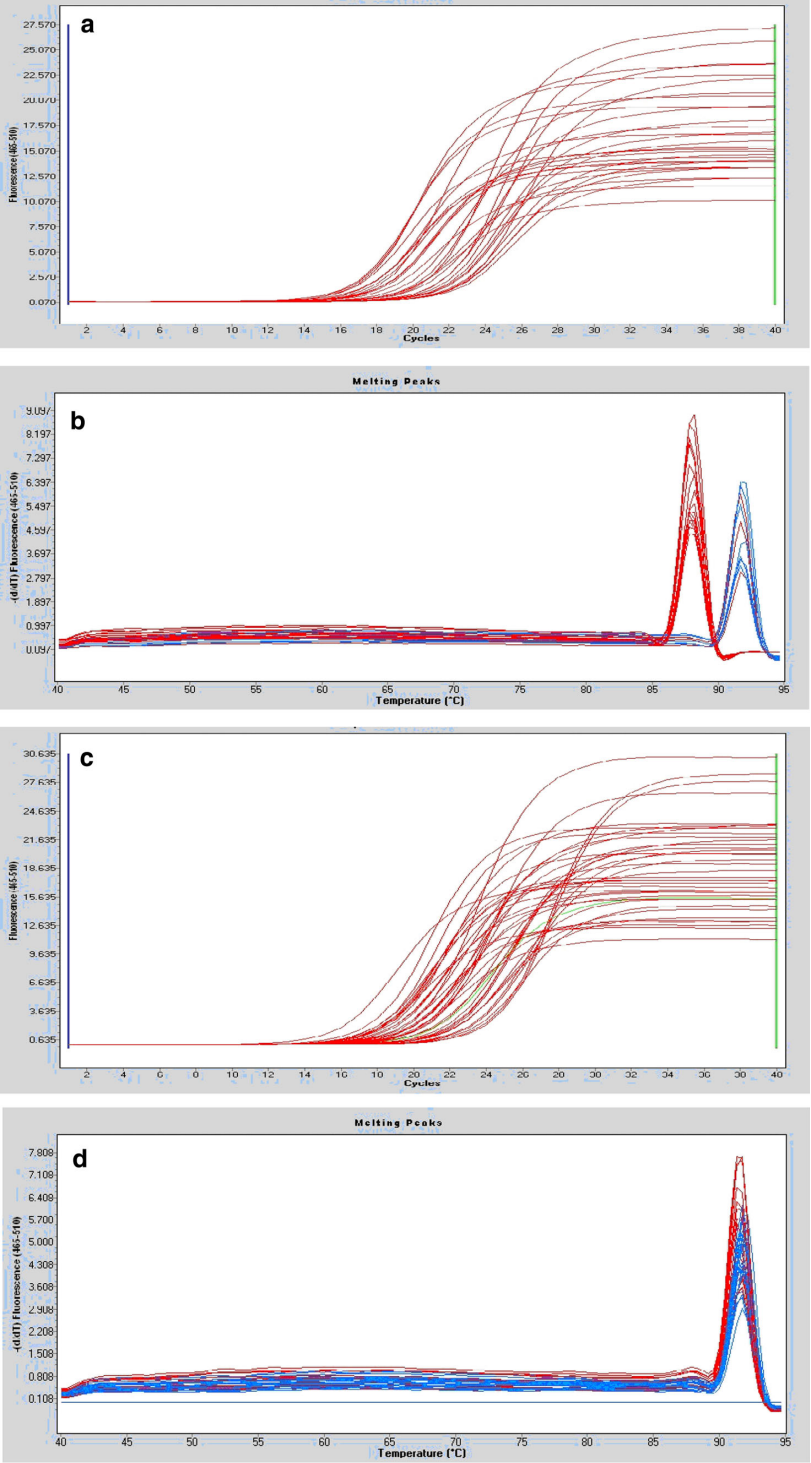

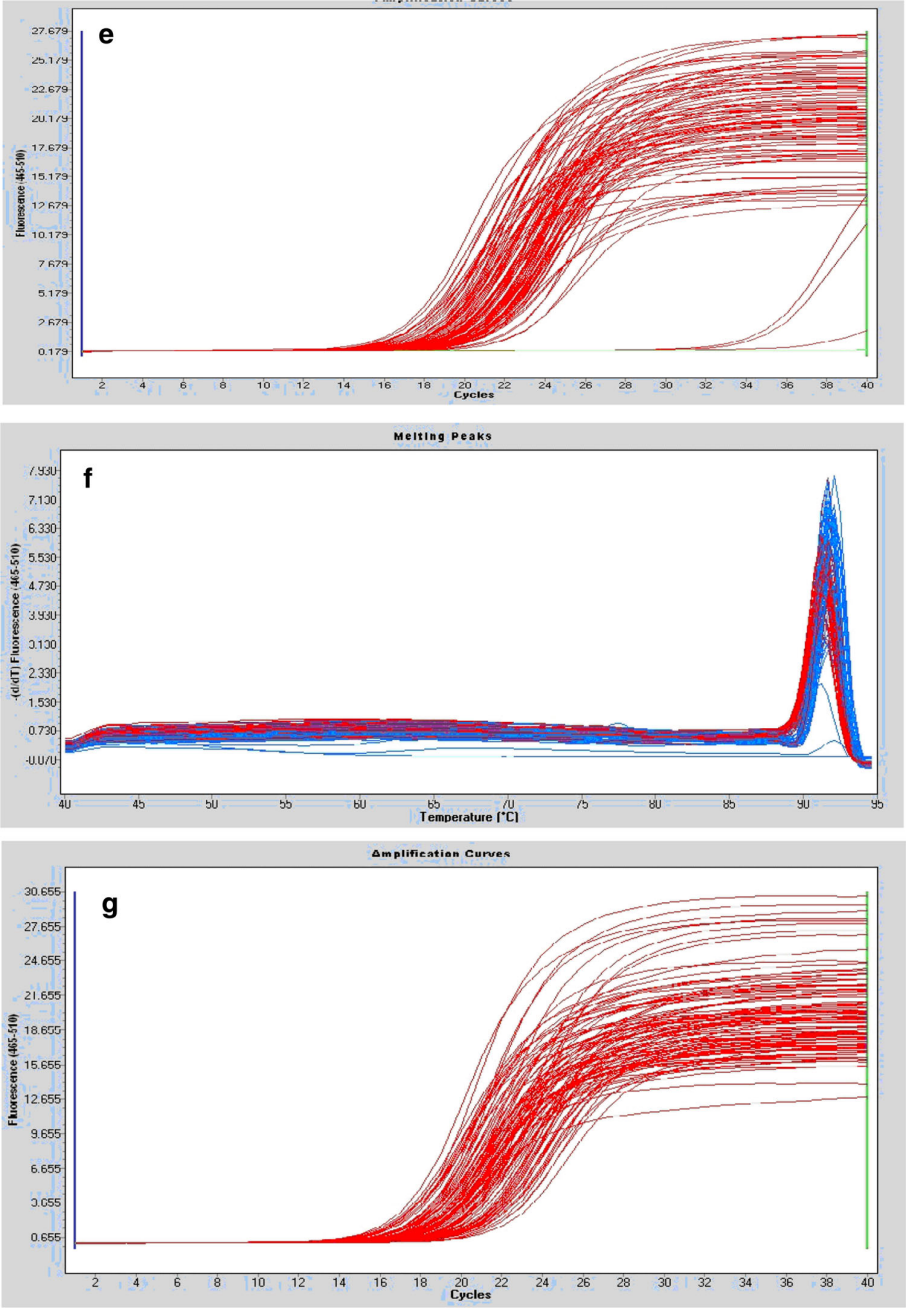

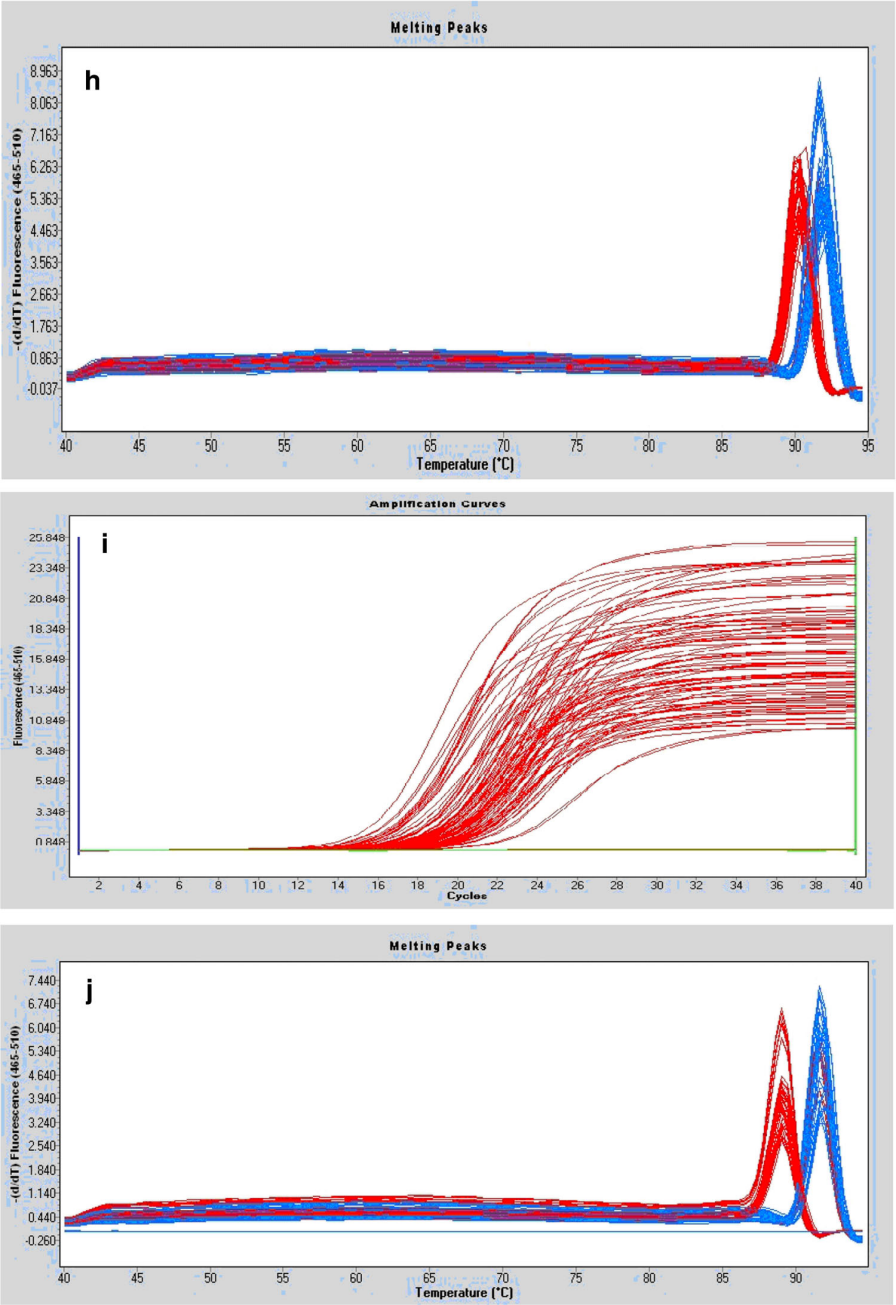
**a** The amplification curves of gene hspX, **b** The melting peaks of gene hspX, **c** The amplification curves of gene hsp65, **d** The melting peaks of gene hsp65, **e** The amplification curves of gene psts1, **f** The melting peaks of gene Psts1, **g** The amplification curves of gene fbpB, **h** The melting peaks of gene fbpB, **i** The amplification curves of gene mpt64, and **j** The melting peaks of gene mpt64

Although the sequences of *hsp*X, *groEL2*, *psts1*, *fbpB,* and *mpt64* were comparable, we considered the possibility that variable expression of these genes or their corresponding proteins might explain the increased intracellular growth rate of the Beijing/W lineage strains. To assess the relative mRNA expression for the different strain types, we performed qRT-PCR. Our results show that *psts1* and *fbpB* were expressed at statistically lower levels in Beijing/W lineage strains [*P* < 0.05; Figs. 1, 2(a–j)].

**Fig. 3:**
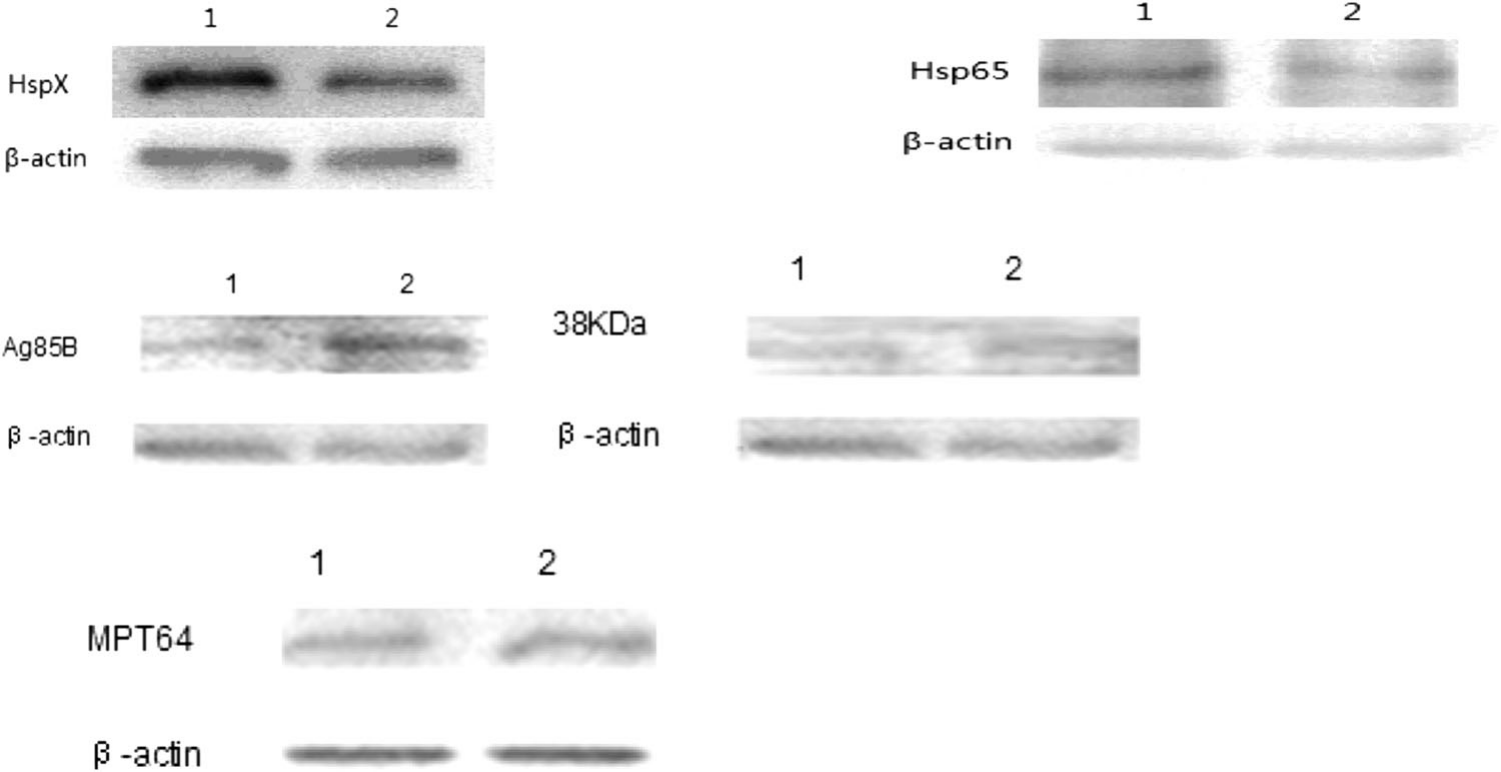
HspX, Hsp65, 38 kDa, Ag85B, and MPT64 protein expression by Western blot analysis *1* Beijing/W, *2* non-Beijing/W

To provide validation for the differences in mRNA expression and to determine whether additional differences in expression could be observed at the protein level, we performed Western blotting for the five corresponding proteins (HspX, Hsp65, 38 kDa, Ag85B, and MPT64) at 24 h after infection of RAW264.7 macrophages. Consistent with the mRNA results, the expression of 38 kDa and Ag85B was lower in the Beijing/W lineage strains than the non-Beijing/W lineage strains. Furthermore, the expression of HspX and Hsp65 was higher in the Beijing/W lineage strains (*P* < 0.05) (Table 5; Fig. 3). These results suggest a mechanism by which the Beijing/W lineage strains may achieve a higher intracellular growth rate.

**Table 5:** The protein expression level in *M*. *tuberculosis*

Lineage	Expression of protein $(\bar{X} \pm S)$				
	hspX	groEL2	psts1	fbpB	mpt64
Beijing/W	2.26 ± 0.23	1.27 ± 0.05	0.89 ± 0.34	0.92 ± 0.23	1.01 ± 0.17
Non-Beijing/W	1.33 ± 0.16	0.92 ± 0.15	1.01 ± 0.22	1.30 ± 0.25	1.04 ± 0.31
H37Rv	1.52 *t* = 32.09	1.03 *t* = 11.23	0.98 *t* = −7.34	1.24 *t* = −13.97	0.93 *t* = −0.89
Compare result	(*P* < 0.05)	(*P* < 0.05)	(*P* < 0.05)	(*P* < 0.05)	(*P* > 0.05)

## Discussion

TB is an ancient contagious respiratory disease. Estimates suggest that one-third of the world’s population is infected with *M. tuberculosis*, while only 5–10 % of those infected will eventually develop active TB. The persistent ability of *M*. *tuberculosis* to mount a stable infection and to adapt to the environment under adverse conditions plays an important role in the occurrence of latent TB infections, as well as in the delay and recurrence of pathogenesis [8]. In the current study, we used Wayne’s hypoxic training model [18] and Betts’s nutrition hungry model [2] to mimic the persistent state of *M. tuberculosis*. We compared the viability of Beijing/W lineage and non-Beijing/W lineage strains and determined that there was no difference between the two groups under any of the host-free culture conditions tested. Subsequently, we compared the viability of the two groups grown intracellularly in macrophages. The two strains initially had similar numbers of viable bacteria within the first 1.5 days after infection. However, over time, the intracellular viability of the Beijing/W lineage strains was significantly more than the viability of the non-Beijing/W lineage strains. On the basis of these findings, we speculate that the prevalence of the Beijing/W lineage strains may be related to their increased ability to survive in macrophages.

*M. tuberculosis* cannot produce endotoxin, exotoxin, or invasive enzymes. Its pathogenicity may be related to its induction of inflammation in tissues in which it multiplies rapidly, to the toxicity of bacterial components and metabolites, or to the immune injury caused by the response to the bacteria. However, proteins of *M. tuberculosis* can also stimulate and induce the host immune system to generate an immune response against *M. tuberculosis*. Given the widespread prevalence of the Beijing/W lineage strains, we speculated that their high transmission and virulence might be associated with genomic variations. However, assessment of 5 major *M. tuberculosis* proteins demonstrated no sequence variations that affect amino acid sequences.

As an alternate possibility, the function of proteins may also be regulated by the level of expression, and we therefore quantified levels of mRNA and protein in Beijing/W and non-Beijing/W lineage strains. Our results show that the expression of *psts1* and *fbpB* and also of their corresponding proteins (38 kDa and Ag85B) is lower for the Beijing/W lineage strains 24 h after infection. 38 kDa is a phosphate transport protein that regulates the efflux of intracellular anti-tuberculosis drugs [3]. Secreted and membrane-bound forms of this protein mediate the apoptosis of macrophages and lead to the upregulation of apoptotic receptors TNFR1, TNFR2, and Fas [14]. Epitopes for T and B lymphocyte recognition within this protein can induce humoral and cellular immunity [19]. Ag85B is a secreted protein that functions within a complex (comprised of Ag85A, B, and C) and has the strongest immunogenicity among the complex components. Ag85B is also the major substance of the BCG effect and is responsible for the induction of Th1 responses [12] and the secretion of IFN-γ from peripheral blood mononuclear cells in healthy people who are PPD positive [16]. The low expression of these two proteins in Beijing/W lineage strains may help Beijing/W strains to escape recognition and evade the immune system, thus contributing to the improved the viability of the bacteria.

We also demonstrated that the expression of HspX and Hsp65 is higher for the Beijing/W lineage strains 24 h after infection. The observed difference was specific to the proteins and was not detected for the associated mRNA, suggesting that the regulation is post-transcriptional. HspX (also called alpha crystal protein) is an important *M. tuberculosis* membrane protein that functions as a molecular chaperone upon heat induction. It is the most highly expressed protein in latent infections and serves as an important target antigen in the body’s immune response to *M. tuberculosis*. HspX plays an important role in gaining entry into the dormant period and in surviving within the in host [7]. This protein is thought to stabilize the structure of the *M. tuberculosis* cell wall and slow bacterial growth [6, 13]. Its upregulation is therefore consistent with the increased viability of Beijing/W lineage strains.

The secreted Hsp65 protein, which showed similar levels of upregulation as HspX, is encoded by the gene gorEL2, is highly conserved, and has strong immunogenicity. Upon entry of *M. tuberculosis* into the host, Hsp65 provides an important antigen that is protective against invasion. In mice infected with TB, about 20 % of the reactive T cells recognize Hsp65 [11]. Given the role of both HspX and Hsp65 in the stress response, variations in their expression could play important roles in the cellular response to *M. tuberculosis*, though the specific mechanism of these proteins in enhancing the viability of Beijing/W lineage strains requires further study.
